# Cytoprotection against Oxidative Stress by Methylnissolin-3-*O*-β-d-glucopyranoside from *Astragalus membranaceus* Mainly via the Activation of the Nrf2/HO-1 Pathway

**DOI:** 10.3390/molecules26133852

**Published:** 2021-06-24

**Authors:** Xiaohua Wu, Jian Xu, Yousheng Cai, Yuejun Yang, Yuancai Liu, Shugeng Cao

**Affiliations:** 1 Department of Pharmaceutical Sciences, Daniel K. Inouye College of Pharmacy, University of Hawai’i at Hilo, Hilo, HI 96720, USA; xiaohua3@hawaii.edu (X.W.); caiyoush@hawaii.edu (Y.C.); 2 Hubei Provincial Key Laboratory of Quality and Safety of Traditional Chinese Medicine Health Food, Jing Brand Research Institute, Jing Brand Co., Ltd., Daye 435100, China; xujian@jingpai.com (J.X.); yyj@jingpai.com (Y.Y.); 3 Institute of TCM and Natural Products, School of Pharmaceutical Sciences, Wuhan University, 185 Donghu Road, Wuhan 430071, China

**Keywords:** *Astragalus membranaceus*, MNG, Nrf2, PI3K, EA.hy926 cells

## Abstract

*Astragalus membranaceus* is a famous herb found among medicinal and food plants in East and Southeastern Asia. The Nrf2-ARE assay-guided separation of an extract from Jing liqueur led to the identification of a nontoxic Nrf2 activator, methylnissolin-3-*O*-β-d-glucopyranoside (MNG, a component of *A. membranaceus*). Nrf2 activation by MNG has not been reported before. Using Western Blot, RT-qPCR and imaging, we investigated the cytoprotective effect of MNG against hydrogen peroxide-induced oxidative stress. MNG induced the expression of Nrf2, HO-1 and NQO1, accelerated the translocation of Nrf2 into nuclei, and enhanced the phosphorylation of AKT. The MNG-induced expression of Nrf2, HO-1, and NQO1 were abolished by Nrf2 siRNA, while the MNG-induced expression of Nrf2 and HO-1 was abated and the AKT phosphorylation was blocked by LY294002 (a PI3K inhibitor). MNG reduced intracellular ROS generation. However, the protection of MNG against the H_2_O_2_ insult was reversed by Nrf2 siRNA with decreased cell viability. The enhancement of Nrf2 and HO-1 by MNG upon H_2_O_2_ injury was reduced by LY294002. These data showed that MNG protected EA.hy926 cells against oxidative damage through the Nrf2/HO-1 and at least partially the PI3K/Akt pathways.

## 1. Introduction

Oxygen is essential for life, but the generation of excess ROS can cause many health problems, such as cancer, rheumatoid arthritis, cardiovascular disease, kidney diseases, respiratory disease, sexual maturation, and neurological disease (e.g., Alzheimer’s disease, Parkinson’s disease, aging and many other neural disorders) [1,2]. The serious threat of oxygen toxicity is often overlooked because all aerobic organisms, including mammals, have intrinsic mechanisms that protect against oxidative damage. To inhibit the oxidative stress that can cause many health problems, one justifiable strategy is to promote the expression of Nrf2 (nuclear factor erythroid 2–related factor 2) and its downstream antioxidant enzymes (e.g., HO-1) [3,4]. The Nrf2 transcription factor, through binding to antioxidant response elements (AREs), induces the expression of a group of downstream anti-oxidant and detoxifying enzymes that protect against oxidative damage and also provide protection against toxic foreign chemical substances through phase II enzyme modification [5,6,7]. Keap1 (Kelch ECH associating protein 1) is a repressor protein that binds to Nrf2 and promotes its degradation by the ubiquitin proteasome pathway [8]. When cells undergoes stress, Keap1 is unable to target Nrf2 due to the change of certain cysteine residues, leading to the phosphorylation and stabilization of Nrf2 in the cytoplasm followed by translocation and accumulation of Nrf2 in the nucleus [9]. In the nucleus, Nrf2 forms a heterodimeric complex with the small Maf (musculoaponeurotic fibrosarcoma) proteins to promote the expression of ARE-mediated antioxidant and detoxifying genes, including heme oxygenase 1 (HO-1), NAD(P)H:quinone oxidoreductase 1 (NQO1), thioredoxin reductase 1 (TrxR1), glutamate-cysteine ligase (GCL), thioredoxin 1 (Trx1) and so on [10]. The phosphoinositide 3-kinase (PI3K)/Akt pathway is another important signaling pathway and its activation could recover the redox balance, defend the oxidative stress, and promote cell survival [11]. It has been reported that the PI3K/Akt pathway could regulate Nrf2 translocation and HO-1 synthesis, and play key roles in regulating Nrf2-ARE-dependent protection [12].

**Figure 1:**
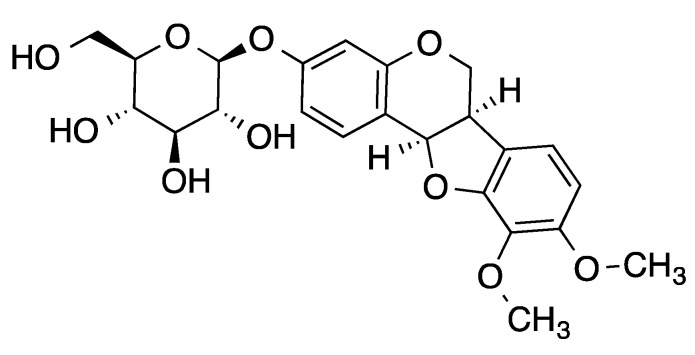
Structure of methylnissolin-3-*O*-β-d-glucopyranoside (MNG, CAS Registry Number 94367–42-7).

Jing liqueur is a beverage containing biologically active compounds from some herbs, including *Astragalus membranaceus,* etc. [13]. In a previous study, we have isolated 189 minor compounds, among which 18 compounds activated Nrf2 at 40 μg/mL in an Nrf2–ARE assay [13]. In our continuing search for Nrf2 activators from the Jing liqueur, we obtained an extract. Nrf2 assay-guided separation led to the isolation of methylnissolin-3-*O*-β-d-glucopyranoside [14] (MNG, Figure 1), a new Nrf2 activator that significantly activated Nrf2 at 40 μg/mL. MNG was much more active than any of the previously isolated 18 Nrf2 activators [13]. MNG was a component of *A. membranaceus*, which is a well-known tonic herb that has been used as both a traditional Chinese medicine and a food or beverage. Traditionally, *A. membranaceus* is primarily used as a lung tonic while its contemporary use mainly focuses on its immunomodulating, antioxidant, and anti-inflammatory, as well as anticancer effects [15]. In 2005, An and his coworkers investigated the neuroprotective roles and direct antioxidant effects of isoflavonoids isolated from the roots of *A. mongholicus* Bunge (Leguminosae) by using the PC12 cell model (a cell line derived from a pheochromocytoma of the rat adrenal medulla) and DPPH (1,1-diphenyl-2-picrylhydrazyl) assay [16], but MNG showed no obvious neuroprotective capability in the PC12 cell-based assay and no apparent scavenging activity against DPPH radicals [16]. In 2014, a research group in Taiwan studied the synergistic antioxidant activity of *A. membranaceus* and *Paeonia lactiflora* and the research group identified 17 antioxidant components, but the activities of the identified compounds including MNG were not investigated [17]. We argue that MNG can enhance the expression of Nrf2, increase the Nrf2 nuclear translocation, activate Nrf2 target proteins, and regulate cytoprotective responses to the stress caused by reactive oxygen species (ROS). We also anticipate that MNG may have some effect on the PI3K/Akt pathway involved in controlling the Nrf2-ARE activity.

In this study, we investigated the effects of MNG on the Nrf2 pathway with human EA.hy926 cells (a type of HUVEC, human umbilical vein endothelial cells), evaluated the protective effect of MNG on the oxidative stress-induced injury of the EA.hy926 cells, and examined whether MNG had an antioxidative capacity through the activation of the PI3K/Akt pathway.

## 2. Results

### 2.1. Identification of MNG as an Nrf2 Activator and Its Cytotoxicity on EA.hy926 Cells

**Figure 2:**
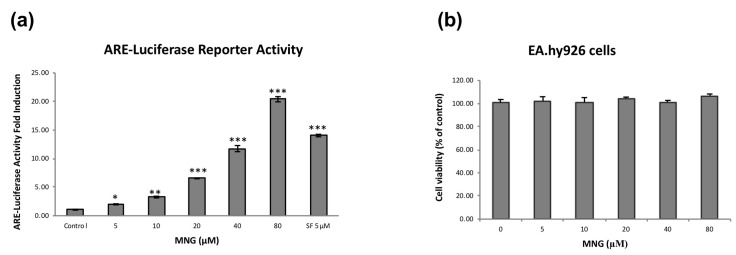
Nrf2 activation and cytotoxicity of MNG. (**a**) The effect of MNG on ARE-luciferase reporter activity in ARE reporter-HepG2 cells. Cells were seeded in 96-well plates at a density of 4 × 10^4^ cells/well and incubated for 24 h. The cells were further treated with 5, 10, 20, 40 and 80 µM of MNG for additional 24 h. The negative control cells were treated with 0.2% DMSO, and positive control cells were treated with 5 μM SF. Luciferase activity was determined. (**b**) The cytotoxicity of MNG toward EA.hy926 cells. Cells were incubated with MNG at the indicated concentrations for 24 h and their viabilities were determined by MTT. The compound had no influence on cell viability at concentrations between 5 and 80 µM for 24 h. The data in the figure represent the mean ± SEM of 3 independent experiments. * *p* < 0.05, ** *p* < 0.01 and *** *p* < 0.001 compared with the control group.

In our previous study, we isolated 189 minor compounds from Jing liqueur [13]. Most of the compounds were assayed on Nrf2–HepG2 stable cells using the luciferase system to identify Nrf2 activators at 40 μg/mL in triplicate [13]. Eighteen compounds were tested active, but they only activated Nrf2 moderately [13]. Further assay-guided separation of a crude extract led to the identification of MNG (3.1 μg/L = 7.7 nM; Figure 1; MS and ^1^H NMR: Figures S1 and S2) as a potent Nrf2 activator. Treatment of Nrf2–HepG2 stable cells with MNG resulted in a dose-dependent increase in ARE-dependent luciferase activity (Figure 2a). The induction of the reporter gene was enhanced about twofold at 5 μM and 20-fold at 80 μM in the luciferase system. Next, we investigated the cytotoxicity of MNG against the EA.hy926 cells with the MTT assay (Figure 2b). No cellular toxicity of MNG was observed at concentrations of up to 80 μM at 24 h. Therefore, in this study, MNG was tested at concentrations between 5 and 80 μM in the subsequent experiments.

### 2.2. MNG Clearly Increased Expression of Nrf2 and Its Target Genes in EA.hy926 Cells

**Figure 3:**
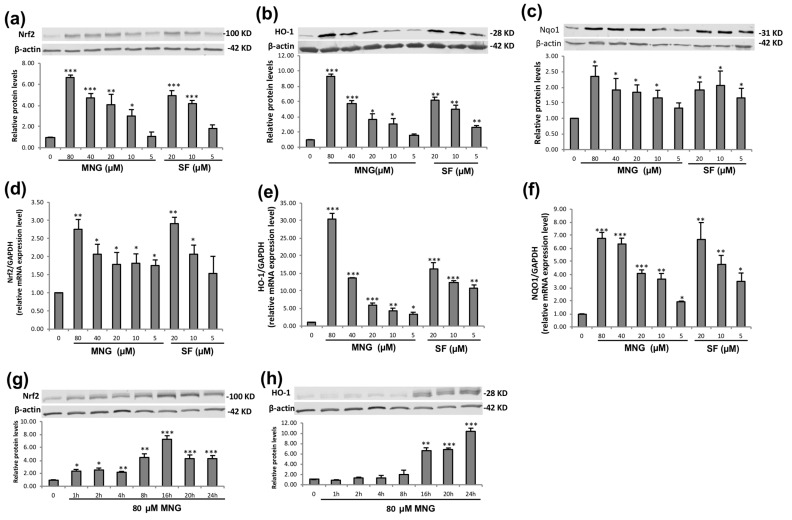
Enhanced expression of Nrf2, HO-1 and NQO1 by MNG in EA.hy926 cells in a dose-dependent manner. (**a**–**c**) The cells were treated for 24 h with MNG and SF at the indicated concentrations. The protein expression of Nrf2, HO-1 and NQO1 was analyzed by Western blot. An equal amount of protein from whole cell lysates was loaded and the β-actin protein level was considered as an internal control. SF was used as a positive control. (**d**–**f**) Total RNA was extracted from EA.hy926 cells treated as indicated. The mRNA expression of Nrf2, HO-1 and NQO1 was determined by real-time qPCR. SF was used as a positive control. (**g**,**h**) EA.hy926 cells were treated with 80 μM MNG for the indicated durations. The Nrf2/HO-1 protein expression was analyzed by Western blot. The data in the figure represent the mean ± SEM of 3 independent experiments. * *p* < 0.05, ** *p* < 0.01 and *** *p* < 0.001 compared with the control group.

To investigate whether MNG could induce the expression of Nrf2-ARE transcriptional activity, we analyzed the effect of MNG on Nrf2 protein and mRNA expressions using Western blot and RT-qPCR, respectively. Similar to the luciferase activity, MNG upregulated the Nrf2 protein expression in a dose-dependent manner (Figure 3a). Since both HO-1 and NQO1 are typical downstream antioxidant enzymes mediated by Nrf2 [18], the effects of MNG on the protein and mRNA expression of HO-1 and NQO1 were also assessed. Results showed that MNG induced the HO-1 (Figure 3b,e) and NQO1 (Figure 3c,f) protein and mRNA expression in a dose-dependent manner. Similarly, expression of Nrf2, HO-1 and NQO1 was strongly induced by the positive control (SF). Western blot analysis was also performed to detect the expression of Nrf2/HO-1 protein as a function of time. The results demonstrated that the MNG treatment significantly increased HO-1 protein expression from 1 h to 24 h in a time-dependent manner (Figure 3h). On the other hand, the MNG treatment resulted in a time-dependent increase in Nrf2 protein expression from 2 h to 16 h. However, the Nrf2 protein expressions at 20 and 24 h treatment times were decreased (Figure 3g).

### 2.3. MNG Effectively Induced Nrf2 Nuclear Translocation in EA.hy926 Cells

**Figure 4:**
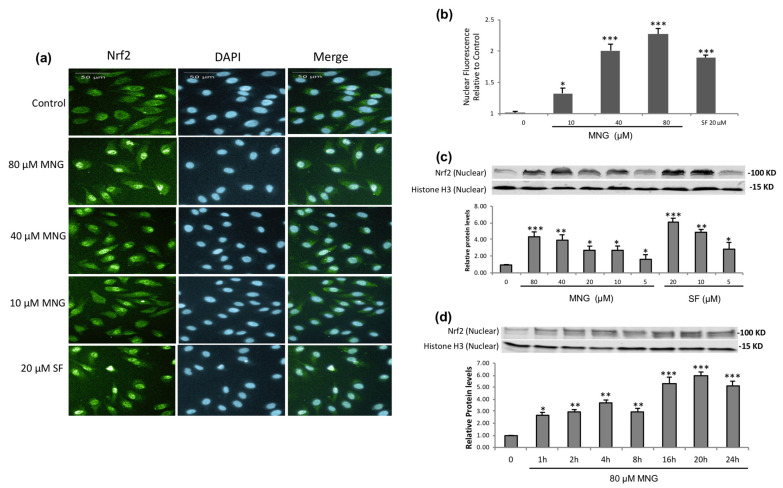
Effects of MNG on nuclear translocation of Nrf2 in EA.hy926 cells. (**a**) Representative images in EA.hy926 cells. Nuclei were stained by DAPI (blue) and transcription factors were stained by immunolabeled antibodies for Nrf2 (green). The fluorescent images were acquired by an Operetta high-content imaging system, using a 20× objective lens (Scale bar represents 50 µm). (**b**) Average relative nuclear fluorescence was quantified by the intensity of fluorescence from individual cells. (**c**) Cells were incubated with MNG and SF at the indicated concentrations for 24 h. The protein expression of Nrf2 in nuclear extracts was examined by Western blot. Histone H3 was used as loading control for nuclear extracts. (**d**) EA.hy926 cells were treated with 80 μM MNG for the indicated durations. The Nrf2 protein translocated into nuclei was analyzed by Western blot. The data in the figure represent the mean ± SEM of 3 independent experiments. * *p* < 0.05, ** *p* < 0.01 and *** *p* < 0.001 compared with the control group.

When exposed to oxidative stress or Nrf2 activators, a few reactive cysteine residues of Keap1 are modified to prevent Nrf2 from proteasome degradation, resulting in the accumulation of Nrf2 in cytosol, followed by rapid nuclear translocation of Nrf2 [19]. To further confirm whether Nrf2 stabilized by MNG was accumulated in the nucleus to stimulate Nrf2-mediated ARE transcription, we then assessed the effect of MNG on the Nrf2 activation via the nuclear enrichment of Nrf2 in EA.hy926 cells. MNG treatment improved the expression of nuclear Nrf2 remarkably, and the peak occurred at 20 h (Figure 4). At 24 h MNG still significantly enhanced Nrf2 nuclear translocation dose dependently with the strongest effect at 80 μM (Figure 4). Fluorescence images from the Operetta imaging system clearly depicted the translocation of Nrf2 to nuclei in response to MNG (Figure 4a). The majority of Nrf2 protein in EA.hy926 cells was localized to nuclei, indicating the activation of the Nrf2 pathway (Figure 4a,b). Western blot analysis indicated that MNG treatment improved the expression of nuclear Nrf2 remarkably and increased Nrf2 abundance in the nuclei dose dependently (Figure 4c). The fidelity of the nuclear preparations was confirmed by Western blot against the nuclear membrane protein Histone H3.

### 2.4. MNG Protected EA.hy926 Cells against H_2_O_2_-Induced Cell Death

**Figure 5:**
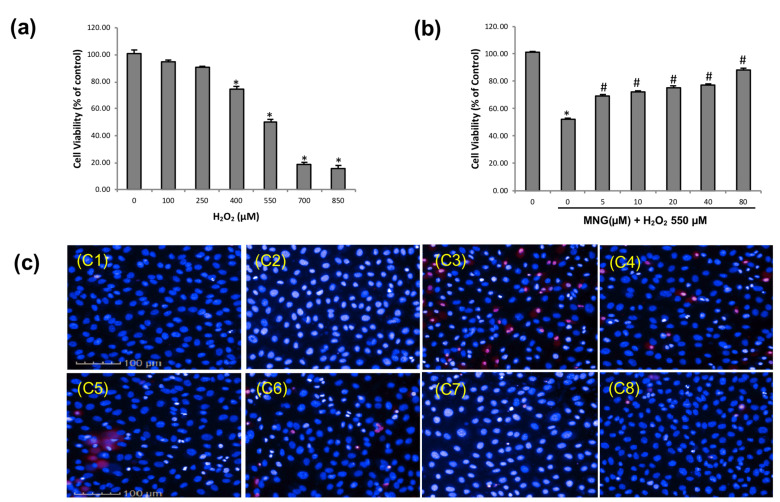
Protection of EA.hy 926 cells by MNG against H_2_O_2_-induced cell death. (**a**) Cells were treated with 550 µM H_2_O_2_ for 6 h. H_2_O_2_ caused a concentration-dependent reduction of cell viability in EA.hy926 cells. (**b**) Cells were pretreated with 0–80 µM MNG for 24 h, followed by incubation with or without 550 µM H_2_O_2_ for 6 h. Cytoprotection of MNG against H_2_O_2_ -induced injury in EA.hy926 cells showed a dose-dependent enhancement from 5 µM to 80 µM. Cell viability was determined by using the MTT assay. * *p* < 0.05 vs. control group, ^#^ *p* < 0.05 vs. 550 µM H_2_O_2_ group. Data are the means ± SEM of 3 independent experiments. (**c**) Cell death was determined by using Hoechst 33,342 and PI double fluorescent staining C1: control; C2: 80 µM MNG; C3: 550 µM H_2_O_2_; C4−C8: 5, 10, 20, 40, 80 µM MNG + 550 µM H_2_O_2_; scale bars represent 100 µm.

Low H_2_O_2_ concentration promotes the beneficial proliferation, but high H_2_O_2_ concentration is deleterious. H_2_O_2_ is a type of ROS that could cross cell membranes and initiate oxidative stress [20]. H_2_O_2_ is also a well-known cell damaging agent and has been used widely in numerous in vitro oxidative stress experiments [21]. To investigate the protection of MNG against cell death induced by ROS, we utilized H_2_O_2_ to induce oxidative injury in EA.hy926 cells in this study (Figure 5). There was a concentration-dependent decrease in cell viability after treatment with H_2_O_2_ (Figure 5a). Compared with cell viability in the control group, H_2_O_2_ at 550 μM caused about 50% decrease in cell viability. Thus, 550 μM H_2_O_2_ was selected for subsequent assays. MNG pretreatment at 5–80 μM attenuated H_2_O_2_-induced cell death in a dose-dependent manner (Figure 5b). To evaluate the cytoprotective effect of MNG, morphologic changes of EA.hy926 cells were measured by using Hoechst 33342/PI double fluorescent staining [22]. Compared with the groups untreated with H_2_O_2_ (Figure 5(c1,c2), control and 80 µM MNG, respectively), cells exhibited the typical features of cell death after treatment with 550 μM H_2_O_2_ (Figure 5(c3)). However, pretreatment with MNG (5, 10, 20, 40 and 80 μM) could effectively protect EA.hy926 cells against H_2_O_2_-induced cell death (Figure 5(c4–c8)).

### 2.5. Inhibition of ROS Production by MNG

**Figure 6:**
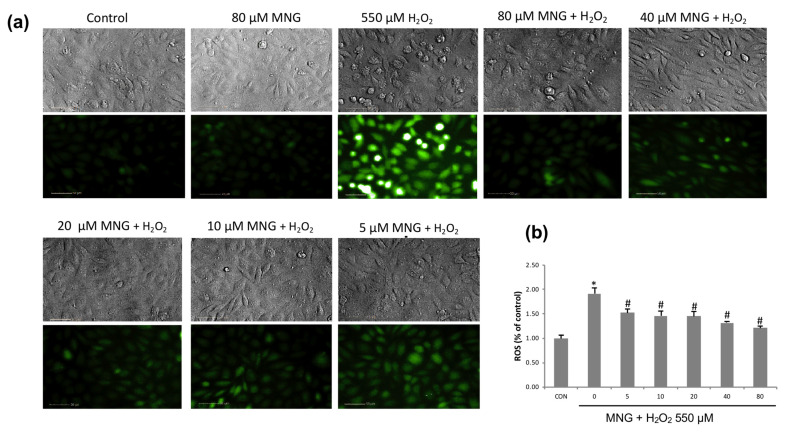
MNG reduced the intracellular ROS generation in EA.hy926 cells. (**a**) Representative images of ROS in EA.hy926 cells were detected by DCFH-DA using the Operetta high content image: vehicle-treated control cells; cells only treated with 80 µM MNG; cells were treated with 0, 5, 10, 20, 40 or 80 µM MNG for 24 h followed by exposure to 550 µM H_2_O_2_ for another 6 h (Scale bars represent 50 µm). (**b**) The levels of intracellular ROS were detected by DCFH-DA using a fluorescence microplate reader. EA.hy926 cells were pretreated with MNG for 24 h at the indicated doses (0, 5, 10, 20, 40 and 80 μM) before treatment with 550 μM H_2_O_2_ for 6 h and then incubated with 10 μM DCFH-DA for 30 min. * *p* < 0.05 vs. control group, ^#^ *p* < 0.05 vs. 550 µM H_2_O_2_ group. Data are the means ± SEM of 3 independent experiments.

Intracellular ROS is an indicator reflecting the level of oxidative stress directly [23]. To investigate the inhibition of ROS production, we investigated the inhibitory effect of MNG on the intracellular ROS generated by H_2_O_2_. Intracellular ROS levels were measured by using a fluorescence microplate reader and an Operetta high-content imaging system after staining with a fluorescent probe, DCFH-DA (Figure 6). Similar to the negative control (Figure 6a, 1st panel), MNG alone did not generate ROS (Figure 6a, 2nd panel). H_2_O_2_ treatment induced a massive production of ROS (Figure 6a, 3rd panel), which caused oxidative stress. However, pretreatment with MNG notably prevented the H_2_O_2_-induced ROS generation in a dose-dependent manner (Figure 6a, panels 4–8). The quantitative intensities of DCFH-DA in various groups measured by using a fluorescence microplate reader are shown in Figure 6b. When stimulated with H_2_O_2_, the intracellular ROS level in EA.hy926 cells increased rapidly. MNG significantly suppressed ROS production in a dose-dependent manner (Figure 6b).

### 2.6. Nrf2 siRNA Attenuated the MNG-Mediated Cytoprotective Effect and Induction of HO-1 and NQO1

**Figure 7:**
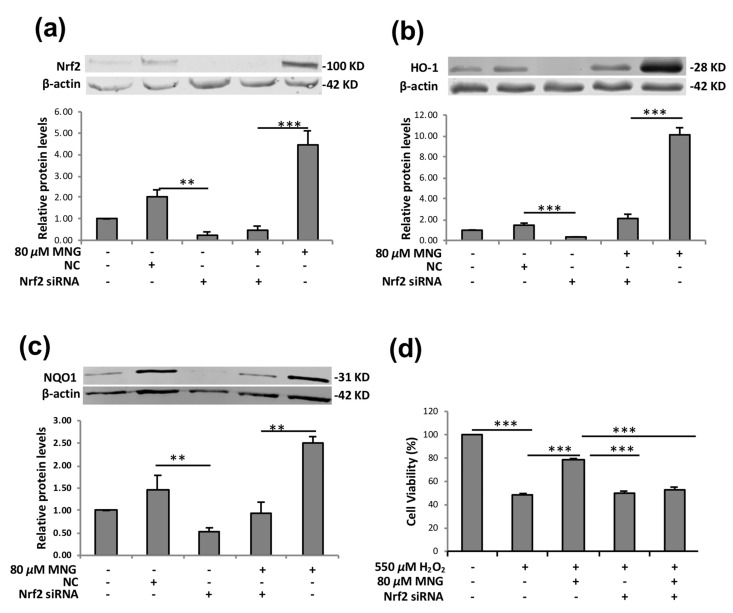
Nrf2 silencing attenuated MNG-mediated cytoprotective effect and induction of HO-1 and NQO1. (**a**–**c**) The cells were transfected with Nrf2 siRNA or NC siRNA, then incubated with or without 80 μM MNG for 24 h, protein expression levels of Nrf2, HO-1 and NQO1 were detected by Western blot. (**d**) Nrf2 silencing reduced the cytoprotective effects of 80 μM MNG on H_2_O_2_-induced cell damage. The cells were transfected with Nrf2 siRNA or NC siRNA, then incubated with or without 80 μM MNG for 24 h, followed by 550 μM H_2_O_2_ for a further 6 h. All data in the figure are presented as means ± SEM of 3 independent experiments. ** *p* < 0.01, *** *p* < 0.001 compared to the indicated group.

The silencing of Nrf2 using siRNA can markedly reduce the Nrf2 level and ARE-driven activity and has been used extensively to study the action mechanisms of Nrf2 activators [24]. To further verify the role of Nrf2 in regulating the expression of its target proteins by MNG, we evaluated the effect of Nrf2 siRNA on the MNG-mediated protective activity against oxidative damage in EA.hy926 cells (Figure 7). Nrf2 siRNA-treated groups showed lower levels of Nrf2 as compared to NC siRNA transfected and non-transfected control cells (Figure 7a). Then, Western blot was employed to further examine the impact of Nrf2 silencing on MNG-mediated induction of HO-1 and NQO1. As shown in Figure 7b,c, Nrf2 silencing significantly suppressed the MNG-induced upregulation of HO-1 and NQO1. An MTT assay revealed that the cytoprotective effects of MNG against the H_2_O_2_ insult were significantly reduced by siRNA-induced knockdown of Nrf2 (Figure 7d).

### 2.7. PI3K/AKT Regulated MNG-Induced Nrf2/HO-1 Expression

**Figure 8:**
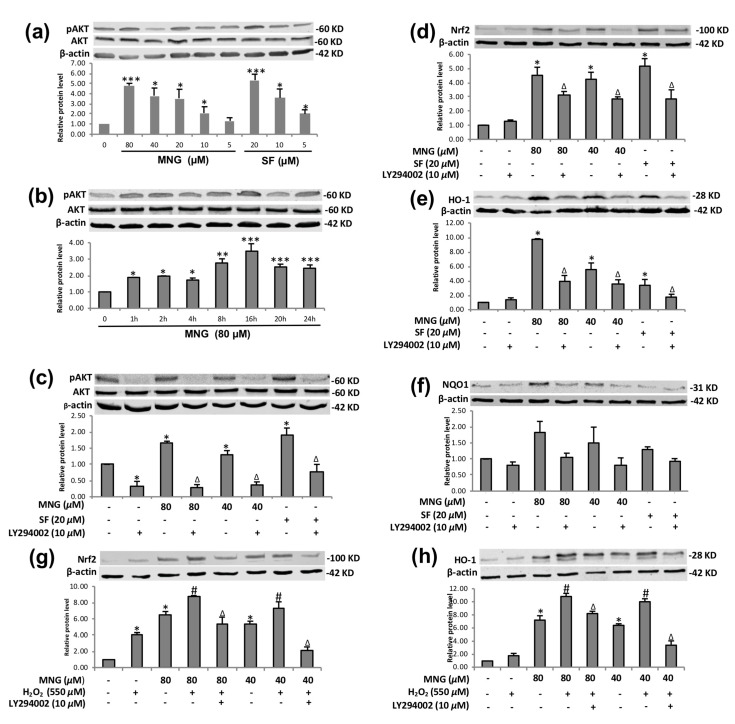
MNG stimulated Nrf2/HO-1 partially via the PI3K/Akt pathway in EA.hy 926 cells. (**a**) EA.hy926 cells were treated with 0–80 μM MNG for 24 h, and the protein levels of p-Akt were determined by using Western blot. (**b**) Cells were treated with 80 μM MNG for various time periods, and the protein levels of p-Akt were determined by using Western blot. (**c**–**f**)**.** After preincubation with 10 μM LY294002 for 2 h, cells were cotreated with 40, 80 μM MNG and 20 μM SF for an additional 24 h, and the protein levels of p-Akt, Akt, Nrf2, HO-1 and NQO1 were then detected by using Western blot. * *p* < 0.05, ** *p* < 0.01, *** *p* < 0.001 vs. control group, ^Δ^ *p* < 0.05 vs. 40, 80 μM MNG or 20 μM SF. (**g**,**h**) After preincubation with 10 μM LY294002 and 40 or 80 μM MNG for 24 h, cells were cotreated with 550 µM H_2_O_2_ for an additional 6 h; the protein levels of Nrf2 and HO-1 were then detected by using Western blot. * *p* < 0.05 vs. control group, ^#^ *p* < 0.05 vs. 550 µM H_2_O_2_ group, ^Δ^ *p* < 0.05 vs. 40, 80 μM MNG + 550 µM H_2_O_2_ group. Data are the means ± SEM of 3 independent experiments.

Studies have demonstrated that the PI3K/Akt pathway acts as an important upstream regulator of Nrf2/HO-1 expression [25], and it is essential in activating the Nrf2-ARE pathway in many types of cells [26,27]. Hence, next, we investigated the possible role of the PI3K/Akt pathway in our study. Treatment with MNG significantly upregulated phosphorylation of Akt in EA.hy926 cells in a dose-dependent manner (Figure 8a). MNG treatment notably enhanced Akt phosphorylation after treatment in a time-dependent manner (Figure 8b), but not the total Akt protein level, suggesting that the PI3K/Akt pathway played a key role in regulating Nrf2-ARE-dependent protection of MNG in EA.hy926 cells.

To further reveal the mechanism, we used LY294002 (a PI3K/Akt inhibitor) to investigate the effect of MNG on the PI3K pathway (Figure 8c–h). The results showed that Akt phosphorylation after MNG treatment was significantly inhibited by LY294002 (Figure 8c), which also attenuated, although did not completely abolish, the MNG-induced Nrf2, HO-1 and NQO-1 activation (Figure 8d, Figure 8e and Figure 8f, respectively).

Furthermore, after preincubation with LY294002 and MNG, EA.hy926 cells were treated with H_2_O_2_. The Western blot results of Nrf2 and HO-1 protein expression showed that LY294002 attenuated the Nrf2 and HO-1 expression of EA.hy926 cells treated by MNG and H_2_O_2_, although not completely blocking the Nrf2 and HO-1 expression. (Figure 8g,h). LY294002 had no obvious inhibitory effect on the NQO-1 expression of EA.hy926 cells treated by MNG and H_2_O_2_ in the experimental setting. These findings further confirmed that to a certain degree, the PI3K/Akt pathway is implicated in the activation of Nrf2/HO-1 by MNG in EA.hy926 cells.

## 3. Discussion

A SciFinder search on June 01, 2021 revealed that there were 117 reports on MNG (CAS Registry Number 94367–42-7) (Figure 1, Figures S1 and S2). MNG was evaluated for its protection of PC12 cells from L-glutamate-induced toxicity, but it was inactive [16]. The inhibitory activity of MNG on LPS-stimulated proinflammatory cytokine production in bone marrow-derived dendritic cells was investigated [28]. The antiaging effect of a formulation containing MNG was also explored [29]. However, none of these 117 publications of MNG was related to Nrf2, indicating that the known compound MNG is a new Nrf2 activator. At the same concentration, SF was more potent than MNG in the ARE-luciferase assay (Figure 2a). However, a further MTT assay showed that MNG did not demonstrate any cytotoxicity against EA.hy926 cells at 500 μM after 24 h, while SF showed certain cytotoxicity at 62.5 μM and killed more than half of the EA.hy926 cells at 250 μM after 24 h (Figure S3), indicating MNG’s superiority to SF when comparing their cytotoxicity, although SF was a more potent Nrf2 activator than MNG. According to the concentration of MNG in the product (7.7 nM), the doses we applied in our experiments were much higher than the content of MNG in the beverage. However, our aims were to investigate the potential mechanisms of action of MNG in protecting EA.hy926 cells against H_2_O_2_-induced oxidative stress through the activation of the Nrf2/HO-1, and to explore the possibility of using MNG as a food additive.

It is interesting that MNG did not increase the level of Nrf2 mRNA at 10 and 20 μM when compared with that at 5 μM (Figure 3d), suggesting that MNG does not activate the Nrf2 pathway constitutively. However, MNG upregulated the Nrf2 mRNA expression slightly at 40 μM and much more at 80 μM (Figure 3d), which might be due to an increased cell reproduction as evidenced by the MTT assay results (Figure S3). When the EA.hy926 cells were treated with MNG, the activation of the Nrf2 protein decreased after 16 h (Figure 3g), suggesting that the physiological response to activate the Nrf2 pathway was already saturated under this condition. The phenomenon was not uncommon [30]. It is possible that Nrf2 reached its maximal activation within 16 h, which might be associated with an overall commitment to Nrf2 activation of the majority of the cells by MNG. After 16 h, probably there was not much remaining Keap1 to be targeted by the electrophiles, resulting in decreased accumulation of Nrf2 in nuclei [31]. It is worth mentioning that there was no significant enhancement of NQO1 protein within 24 h (Figure 3a–c) when compared with Nrf2 and HO-1, suggesting that it might take a longer time for MNG to increase the NQO1 protein in EA.hy926 cells. However, MNG enhanced the expression of NQO-1 in a dose-dependent manner (Figure 3c). Nevertheless, the results strongly support the hypothesis that MNG could enhance the cytoprotective effect and antioxidant response through activating Nrf2 and then inducing the expression of HO-1 and NQO1 expression.

The well-known Nrf2 activator, resveratrol induces the translocation of Nrf2 to nuclei in different types of cell lines [32]. As an electrophile, SF (an Nrf2 activator, widely used as a positive control) also enables Nrf2 to escape from Keap1-dependent degradation, which induces the nuclear translocation of Nrf2 followed by the activation of phase 2 enzymes in EA.hy926 cells [33]. The results were in agreement with our study (Figure 4), in which both SF- (positive control) and MNG-induced nuclear translocation of Nrf2 was confirmed by Operetta imaging and Western blot. A time course study showed that there was an accumulation of Nrf2 in the nucleus shortly after MNG treatment, but that there was more Nrf2 nuclear translocation at 16–24 h (Figure 4d and Figure S4). The time course pattern of the Nrf2 nuclear translocation after MNG treatment was similar to the time-dependent pattern of the Nrf2 protein expression as shown in Figure 3g. Hence, MNG might stabilize Nrf2 protein in the cytosol and then enhance its translocation to the nuclei.

Many Nrf2 activators, such as resveratrol, showed an inhibitory effect against the harmful effect of ROS [34]. Our results (Figure 5) demonstrated that MNG could also inhibit the deleterious effect of ROS, exerting its cytoprotective activity by blocking cell death and recovering cell viability in oxidative-stress-stimulated EA.hy926 cells, which was further supported by the experimental results as shown in Figure 6.

It was reported that Nrf2 activators could suppress the production of ROS. For example, pterostilbene (a 3,5-O-dimethylated resveratrol) could restore vascular redox balance, which was evidenced by decreased H_2_O_2_ production [35]. MNG could also inhibit the production of H_2_O_2_-induced intracellular ROS in EA.hy926 cells, indicating that MNG had an antioxidative effect. Meanwhile, MNG alone could not induce the oxidative stress in EA.hy926 cells because the MNG treatment alone had no effect on the level of intracellular ROS (Figure 6a, 2nd panel). The data strongly suggested that MNG could enhance the defensive system of endogenous antioxidation, which has been proven to be the frontline of defense against oxidative stress [36,37]. The result was further confirmed by the abolishment of the MNG’s protection against H_2_O_2_ insult by Nrf2 siRNA (Figure 7).

MNG could protect EA.hy926 cells from H_2_O_2_ damage. However, when the Nrf2 expression was downregulated by siRNA, the EA.hy926 cells were susceptible to H_2_O_2_ insult both in the presence and absence of MNG (Figure 7d), indicating that MNG exerted its protective effect against H_2_O_2_ through the activation of Nrf2; otherwise, siRNA could not block the cytoprotective effect of MNG against H_2_O_2_ insult. These results suggest that Nrf2 is an essential factor for the MNG-induced ARE transcriptional activity, including the expression of Nrf2′s downstream genes (for examples, HO-1 and NQO1). Clearly, the MNG-induced cytoprotective effect against H_2_O_2_ was mediated by the activation of Nrf2 in EA.hy926 cells.

The pAkt/Akt ratio in Figure 8b exhibited a broad bell-shape [38], and reached its maximal activation by MNG within 16 h. This might also be associated with an overall commitment to Akt activation of the majority of the cells by MNG. After 16 h, probably there was not much remaining Akt to be phosphorylated, resulting in the decreased expression of pAkt. The results suggested that MNG could enhance the cytoprotective effect and antioxidant response through activating PI3K/Akt pathway, at least to a certain extent. If the activation of Nrf2 by MNG were regulated solely through the activation of the PI3K/Akt pathway, LY294002 should be able to completely block the activation of Nrf2, HO-1 and NQO1 by MNG (Figure 8d–f). Therefore, we concluded that PI3K/Akt pathway is important for the cytoprotection of MNG, and the pathway is partially involved in the activation of the Nrf2/HO-1 (NQO1) pathways by MNG, which was further supported by the partial abolishment of the MNG’s protection against H_2_O_2_-induced insult by LY294002 as shown in Figure 8g,h. It is worth mentioning that under certain concentrations, H_2_O_2_ can increase the Nrf2 protein through the de novo synthesis of Nrf2, as evidenced in Figure 8g,h, but a high concentration of H_2_O_2_ is toxic as shown in Figure 5a.

Taken together, our results show for the first time that the new Nrf2 activator MNG produced by *A. membranaceus* and isolated from Jing liqueur could attenuate the H_2_O_2_-induced cell death and oxidative stress in EA.hy926 cells, which may be involved in the activation of the Nrf2/HO-1 and partially the PI3K/Akt pathways. Our results may also provide certain scientific support to the use of MNG as a food additive. Perhaps other signaling factors (for example, p38, MAPK, JNK, and GSK-3*β*) are involved in the Nrf2 expression induced by MNG, which are worthy of further investigation. It is also worth carrying out an in vivo study, investigating the effects and mechanisms of MNG on the other types of cells, such as human neuroblastic and beta cells and so on, and studying its preventive effects on chronic diseases, such as cancer, Alzeimer’s, diabetic and cardiovascular diseases.

## 4. Materials and Methods

### 4.1. Materials

TRIzol reagent (#T9424), radioimmunoprecipitation assay buffer (RIPA buffer, #R0278) and Tris-buffered saline containing 0.1% Tween-20 (TBST) were obtained from Sigma (St. Louis, MO, USA). Primary antibodies against Nrf2 (#ab62352) and NQO1 (#ab34173) were purchased from Abcam (Cambridge, MA, USA). HO-1 (#5853S), p-Akt (#4060S), Akt (#4691S), β-actin (#3700S), and Histone H3 (#4499S) were purchased from Cell Signaling Technology (Danvers, MA, USA). Second antibody IRDye 680RD goat anti-mouse IgG (#926–68070) and IRDye 800CW goat anti-rabbit IgG (#926–32211) were purchased from LI-COR, Inc. (Lincoln, NE, USA). NE-PER Nuclear and Cytoplasmic Extraction Kit (#78835) and Pierce BCA Protein Assay Kit (#23227) were purchased from Thermo Fisher Scientific (Hanover Park, IL, USA). Nitrocellulose membrane, iTag universal SYBR green supermix (#1725124) and iScript cDNA synthesis kit (#1708891) were obtained from Bio-Rad (Hercules, CA, USA). Nrf2 Antioxidant Pathway ARE Reporter–HepG2 cell line (#Q16236) was purchased from BPS Bioscience (San Diego, CA, USA). EA.hy926 cells (#83–0020) were obtained from the UNC, Lineberger Comprehensive Cancer Center (Chapel Hill, NC, USA). Eagle’s minimum essential medium (EMEM) and Dulbecco’s Modified Eagle Medium (DMEM) were bought from Corning (New York, NY, USA). Fetal bovine serum (FBS) was purchased from Invitrogen (Waltham, MA, USA). RNeasy Mini Kit (#74106) was bought from Qiagen (Hilden, Germany). Small interference RNAs (siRNA) against Nrf2 (siNrf2, #sc-37030) as well as control-siRNA (c-siRNA, #sc-37007) were purchased from Cruz Biotechnology (Santa Cruz, CA, USA).

### 4.2. Chemicals

Compounds were dissolved as a 40 mM stock solution in dimethyl sulfoxide (DMSO), stored at −20 °C, and diluted to test concentrations with culture medium immediately prior to the experiment. The final concentration of DMSO in the culture medium was less than 0.2%. Hydrogen peroxide (H_2_O_2_), DMSO, L-sulforaphane (SF, #S6317), 3-(4,5-dimethylthiazol-2-yl)-2,5-diphenyltetrazolium bromide (MTT, #M5655), propidium iodide (PI, #81845) and the PI3K inhibitor LY294002 (#440204) were purchased from Sigma (St Louis, MO, USA). Penicillin, streptomycin and 4′,6-diamidino-2-phenylindole (DAPI, #62247) were purchased from Thermo Fisher (Waltham, MA, USA), while Hoechst 33,342 (#H3570), DCFH-DA (2′-7′dichlorofluorescin diacetate, #D399) and lipofectamine 2000 transfection reagent (#13778030) were also bought from Thermo Fisher but at a different site (Hanover Park, IL, USA). MNG (#QP-1913) was purchased from Quality Phytochemicals LLC (East Brunswick, NJ, USA).

### 4.3. Cell Culture

Cells were cultured according to manufacturers’ instructions. The ARE Reporter−HepG2 cell line contains a firefly luciferase gene under the control of ARE stably integrated into HepG2 cells. This cell line is validated for the response to the stimulation of *tert*-butylhydroquinone (TBHQ) and SF, and is used to monitor Nrf2 antioxidant response pathway activity and screen for activators or inhibitors of the Nrf2 antioxidant response pathway. Nrf2-ARE–HepG2 cells were cultured at 37 °C in a humidified incubator with 5% CO_2_, in EMEM with nonessential amino acids and supplemented with 10% FBS, penicillin and streptomycin. Cells were selected with the aminoglycoside antibiotic, G418 (500 μg/mL) and split every 4 to 5 days. EA.hy926 cells were cultured in DMEM supplemented with 10% heat-inactivated FBS, penicillin (100 U/mL), and streptomycin (100 U/mL) at 37 °C in a humidified atmosphere containing 95% air and 5% CO_2_. All cells were from <10 passages.

### 4.4. Luciferase Reporter Gene Assay

The Nrf2-ARE–HepG2 stable cell line was seeded into 96-well plates at 4 × 10^4^ per well in a final volume of 100 µL MEM. Medium was replaced with fresh MEM after seeding for 24 h and the cells were treated with or without MNG (5–80 μM). Plates were incubated for 24 h, then 100 µL ONE-Step Luciferase reagent (BPS Bioscience) was added to each well and the assay was performed according to manufacturer’s instructions. Luminescence was detected using a luminometer (LUMIstar Galaxy BMG, Offenburg, Germany) and data are expressed as relative luminescence units (RLU) emitted from total assays. SF (5 μM) was used as a positive control [13].

### 4.5. Cell Viability Assay 

An MTT assay was performed to estimate cell viability. EA.hy926 cells (1 × 10^4^ per well) were seeded in 96-well plates, cultured at 37 °C in a 5% CO_2_ incubator for 24 h, and then incubated with or without MNG (5–80 μM) for 24 h. For induction of oxidative stress, cells were exposed to H_2_O_2_ at 0, 100, 250, 400, 550, 700 and 850 μM for 6 h. Compared with cell viability in the control group, H_2_O_2_ at 550 μM caused about 50% decrease in cell viability. Thus, H_2_O_2_ at the concentration of 550 μM was chosen for the follow-up experiments_._ To test cytoprotective effect of MNG, EA.hy926 cells were first treated with indicated concentration of MNG, then incubated with H_2_O_2_ (550 μM) for 6 h. 20 μL of MTT (5 mg/mL) in PBS was added to each well, and cells were further incubated at 37 °C for 4 h. The culture medium was carefully removed, and 200 μL of DMSO was added per well to dissolve the formed precipitate. Plates were shaken for 10 s, and absorbance was measured at the wavelength of 570 nm on a microplate reader (BIO-TEK instruments, Inc., Winooski, VT, USA) [39,40].

### 4.6. Hoechst 33342/PI Fluorescent Staining 

EA.hy926 cells were plated at 1.5 ×10^4^ cells/well in 96-well microplates (Greiner Bio-One, Frickenhausen, Germany) and incubated for 24 h, and then the culture medium was removed. Compounds were prepared at various concentrations in 1% FBS with phenol red-free DMEM and incubated for 24 h. A sample of 550 μM H_2_O_2_ diluted in 1% FBS with phenol red-free DMEM was treated for 6 h for induction of oxidative stress. Cytotoxicity was measured by using Hoechst 33,342 and PI co-staining. Cells were stained with 2 μg/mL Hoechst 33,342 (excitation/emission of 350 nm/461 nm) for 30 min and 2 μg/mL PI (excitation/emission of 535/617 nm) for 15 min in the dark. PI stains nuclei of dead cells, whereas Hoechst33342 stains nuclei of all cells. The fluorescent images were acquired by using an Operetta high-content imaging system (Perkin Elmer, Waltham, MA, USA) [30,41].

### 4.7. ROS Detection 

To monitor the intracellular accumulation of ROS, the fluorescence-generating probe DCFH-DA was used. Briefly, EA.hy926 cells seeded in 96-well plates at the density of 1.5 × 10^4^ per well were cultured overnight. The cells were pretreated with various concentrations of MNG for 24 h followed by exposure to 550 μM H_2_O_2_ for 6 h. After washing with PBS twice, cells were incubated with a serum-free medium containing 10 μM DCFH-DA at 37 °C for 30 min, and then washed with PBS twice to remove extracellular DCFH-DA. Intracellular ROS were measured immediately using a microplate reader (BIO-TEK instruments, Inc., Winooski, VT, USA). Fluorescence staining was visualized on an Operetta high-content imaging system (PerkinElmer, Waltham, MA, USA) [30].

### 4.8. Isolation of Nuclear and Cytoplasmic Extract

The cells (1 × 10^6^ cells per well in 60 mm dishes) were treated with MNG at various concentrations (0, 5, 10, 20, 40 and 80 µM) and SF (5, 10 and 20 µM) for 24 h. The nuclear extraction was prepared using an NE-PER Nuclear and Cytoplasmic Extraction Reagent kit according to the manufacturer’s instruction. The resulting supernatant, constituting the cytoplasmic and nuclear extract, was used for the subsequent experiments. The protein concentrations were determined by BCA protein assay (bicinchoninic acid protein assay).

### 4.9. Western Blot Analysis 

Total protein was extracted from the cells and prepared with RIPA buffer. Nuclear or cytoplasmic proteins were isolated as described above. Protein samples were boiled for 5 min at 95 °C. The protein concentrations were quantified by the BCA protein assay. In brief, samples with the same amount of proteins were separated on 10% polyacrylamide gel. The proteins were then electrophoretically transferred at 15 V at RT onto a NC membrane. The blotted membranes were blocked in 5% nonfat dry milk in TBST for 1 h and then incubated overnight at 4 °C with the indicated primary antibodies. After three washes with TBST, the membranes were then incubated with corresponding secondary antibodies for 1 h and bands were visualized and analyzed by the LI-COR odyssey image system (Lincoln, NV, USA). Each Western blot analysis was performed in triplicate [42].

### 4.10. Real-Time PCR Analysis 

Total RNA was extracted from the treated cells (1 × 10^6^ cells per well in 60 mm dishes) by using the TRIzol reagent and RNeasy Mini Kit. cDNA was prepared with iScript cDNA synthesis kit. Multi-gene transcriptional profiling with quantitative RT-PCR was performed with iTag universal SYBR green supermix and Bio-Rad CFX96 real-time system. All reactions were performed in triplicate. The relative expression of target genes was normalized to the expression of housekeeping gene GAPDH, calculated by the 2^−ΔΔCT^ method and given as ratio compared with control. Quantitative real-time PCR was performed using the following primers: Nrf2, F: 5′-GAGACAGGTGAATTTCTCCCAAT-3′, R: 5′-TTTGGGAATGTGGGCAAC-3′; HO-1, F: 5′-GCTGAGTTCATGAGGAACTTTCAG-3′, R: 5′-TGGTACAGGGAGGCCATCAC-3′; NQO1, F: 5′-ATGTATGACAAAGGACCCTTCC-3′, R: 5′-TCCCTTGCAGAGAGTACATGG-3′; GAPDH, F: 5′-GAGCCAAAAGGGTCATCATC-3′, R: 5′-TAACAGTTGGTGGTGCAGG-3′ [41,43].

### 4.11. Immunofluorescence Analysis 

EA.hy926 cells were seeded at 1 × 10^4^ cells/well in 96-well microplates overnight and then treated with various concentrations of MNG or SF. After 24 h, the cells were fixed with 4% paraformaldehyde for 30 min followed by 0.3% Triton to punch at RT for 30 min. After washing 3 times with PBS buffer and blocking with 5% BSA for 1 h, the cells were incubated with a primary antibody of anti-Nrf2 (1:500) overnight at 4 °C followed by incubation of an Alexa Fluor 488-conjugated goat anti-rabbit monoclonal fluorescent secondary antibody (1:1000) for 1 h at RT. After washing three times with PBS, the cells were stained with DAPI for 15 min. The plate was scanned by the Operetta high-content imaging system (PerkinElmer), and the Harmony software (version 4.1) was used to calculate the percentage of nuclear translocation [44].

### 4.12. RNA Interference of Nrf2 

Transient transfection of siRNA was performed with the lipofectamine 2000 transfection reagent according to the manufacturer’s instructions. In brief, the cells were seeded in a 6-well culture plate (3 × 10^5^ cells per well) to reach 60% confluency and transfected with negative control (NC) siRNA or Nrf2 siRNA. The transfected cells were prepared and the expressions of Nrf2 and target genes were analyzed [45].

### 4.13. Statistical Analysis

Unless otherwise mentioned, all data exhibited in the study were expressed as mean ± SEM from at least 3 independent experiments, each in triplicate for individual treatment or dosage. Statistical differences between two groups (usually the control and an experimental group) were evaluated by *t*-test. One-way ANOVA was used to evaluate the differences between multiple groups, and *p* < 0.05 was considered as statistical significance.

## Data Availability

Data sharing not available.

## Supplementary Materials

The following are available, Figure S1: MS spectrum of MNG, Figure S2: ^1^H NMR spectrum (400 MHz) of MNG in DMSO-*d*_6_, Figure S3: Cytotoxicity of MNG (left) and SF against EA.hy926 cells.

## Sample Availability

Samples generated from Jing Liqueur but not the pure compounds are available from the authors.

## Abbreviations and Nomenclature

Antioxidant response element (ARE); bicinchoninic acid assay (BCA assay); 4′,6-diamidino-2-phenylindole (DAPI); 2′-7′-Dichlorofluorescin diacetate (DCFH-DA); Dulbecco′s modified eagle medium (DMEM); dimethyl sulfoxide (DMSO); 1,1-Diphenyl-2-picrylhydrazyl (DPPH); Eagle′s minimum essential medium (EMEM); EA.hy926: a type of human umbilical vein endothelial cell line; fetal bovine serum (FBS); glutamate-cysteine ligase (GCL); Heme oxygenase 1 (HO-1), human umbilical vein endothelial cells (HUVEC); Kelch ECH associating protein 1 (Keap1); musculoaponeurotic fibrosarcoma (Maf); methylnissolin-3-*O*-β-d-glucopyranoside (MNG); 3-(4,5-dimethylthiazol-2-yl)-2,5-diphenyltetrazolium bromide (MTT); negative control (NC) membrane; NAD(P)H:quinone oxidoreductase 1 (NQO1); nuclear factor erythroid 2–related factor 2 (Nrf2); PCl12: a cell line derived from a pheochromocytoma of the rat adrenal medulla; propidium iodide (PI); phosphoinositide 3-kinase (PI3K); radioimmunoprecipitation assay buffer (RIPA buffer); relative luminescence units (RLU); reactive oxygen species (ROS); small interference RNAs (siRNA); L-sulforaphane (SF); tert-butylhydroquinone (TBHQ); tris-buffered saline containing 0.1% tween-20 (TBST); thioredoxin 1 (Trx1); thioredoxin reductase 1 (TrxR1).
