# Supplementary material for: Cytoprotection against Oxidative Stress by Methylnissolin-3-O-β-d-glucopyranoside from Astragalus membranaceus Mainly via the Activation of the Nrf2/HO-1 Pathway
Source: Molecules. 2021 Jun 24;26(13):3852. doi: 10.3390/molecules26133852 (PMC8270303; doi:10.3390/molecules26133852)
Supplement: Supplementary file 1 [file molecules-26-03852-s001.zip › molecules-1243266-supplementary.pdf]

Supplementary material

Cytoprotection against Oxidative Stress by methylnissolin-3-O-β-D-glucopyranoside from *Astragalus membranaceus* mainly via the Activation of the Nrf2/HO-1 Pathway

Xiaohua Wu [a], Jian Xu [b], Yousheng Cai [a,c], Yuejun Yang [b], Yuancai Liu [b,*], Shugeng Cao,[a,*]

[a] Department of Pharmaceutical Sciences, Daniel K. Inouye College of Pharmacy, University of Hawai'i at Hilo, Hilo, Hawaii 96720, United States

[b] Hubei Provincial Key Laboratory of Quality and Safety of Traditional Chinese Medicine Health Food, Jing Brand Research Institute, Jing Brand Co., Ltd., Daye 435100, China

[c] Institute of TCM and Natural Products, School of Pharmaceutical Sciences, Wuhan University, 185 Donghu Road, Wuhan 430071, China

*Corresponding Author

Shugeng Cao: E-mail: scao@hawaii.edu; Tel: 1-808-981-8010; Fax: 1-808-933-2974; ORCID number: 0000-0001-6684-8221

Fig. S1: MS spectrum of MNG

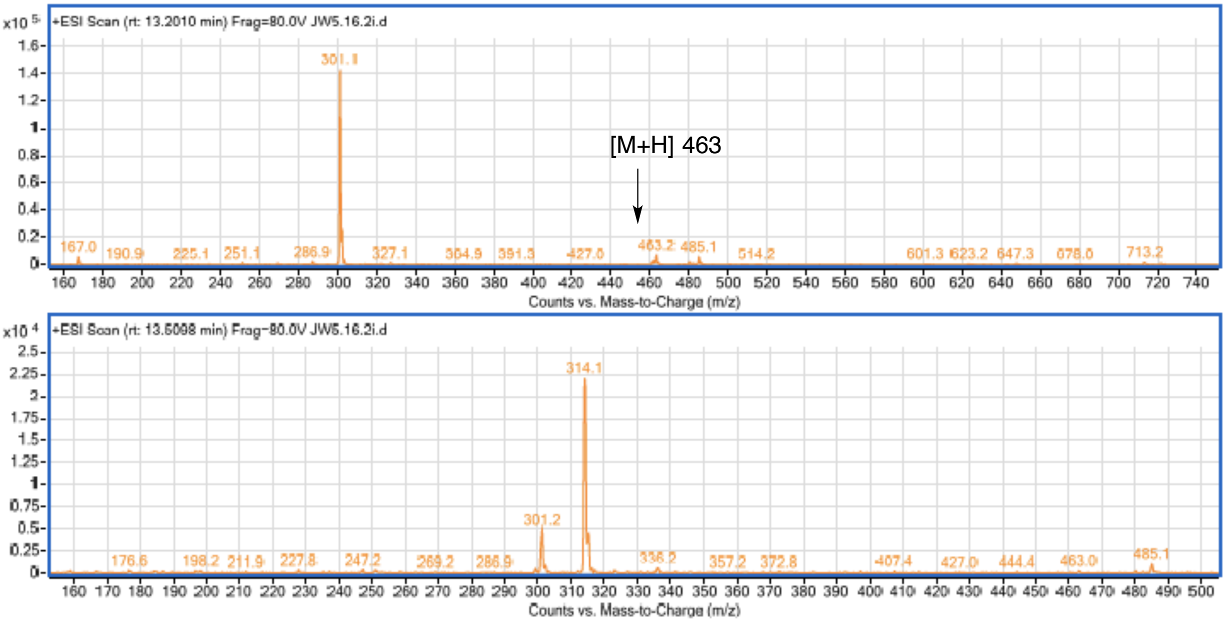

Fig. S2: ^{1}H NMR spectrum (400 MHz) of MNG in DMSO-d_6

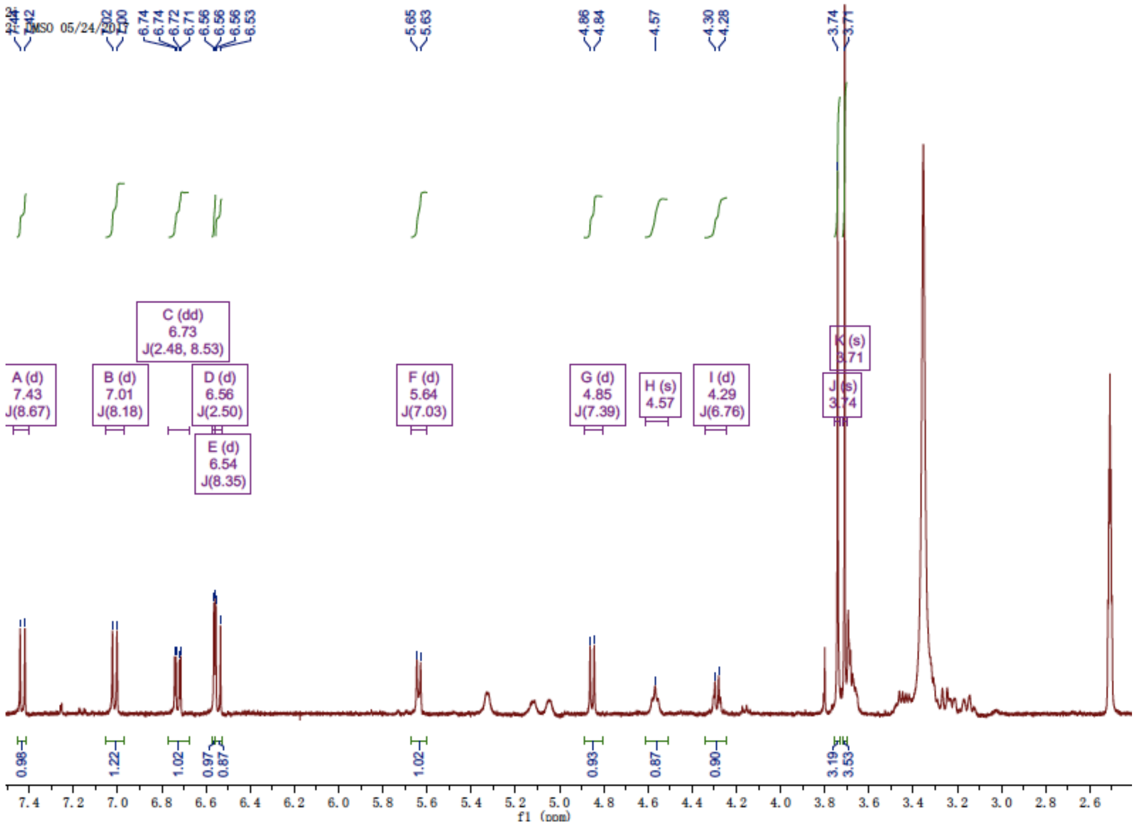

Fig. S3: Cytotoxicity of MNG (left) and SF against EA.hy926 cells

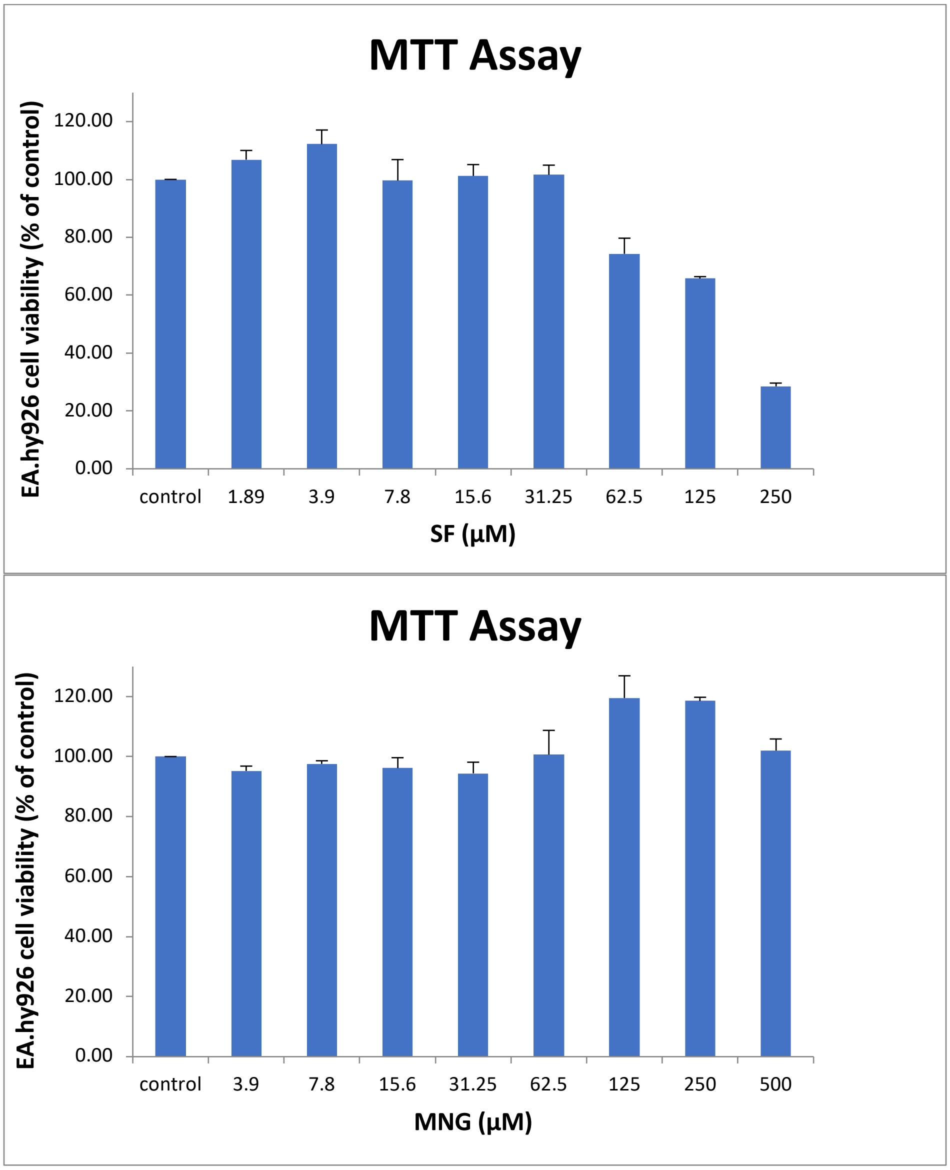

The cytotoxicity of MNG and SF (L-sulforaphane) toward Ea.hy926 cells. Cells were seeded in 96-well plates at a density of 1×10^4 cells/well and incubated for 24 h. Cells were further incubated with MNG and SF at the indicated concentrations for 24 h and their viabilities were determined by MTT. The MNG compound had no influence on cell viability at concentrations between 3.9 µM and 500 µM for 24 h. SF started to show the cytotoxicity from 62.5 µM, and killed more than 50% of cells at 250 µM.

Fig. S4: Effects of 80 μM MNG on nuclear translocation of Nrf2 in EA.hy926 cells.

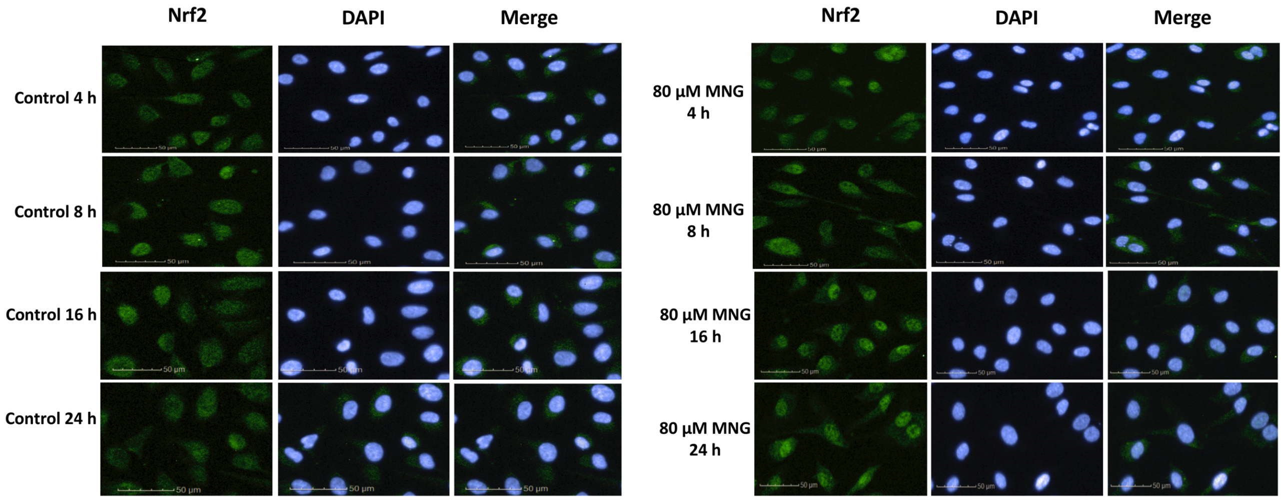

Representative images in EA.hy926 cells. Nuclei were stained by DAPI (blue) and transcription factors were stained by immunolabeled antibodies for Nrf2 (green) at different time points (4, 8, 16 and 24 h) after 80 μM MNG treatment. 0.2% DMSO was used as a positive control. The fluorescent images were acquired by an Operetta high-content imaging system using a 20× objective lens (Scale bar represents 50 μm).
